# Prophylactic vaccines are potent activators of monocyte-derived dendritic cells and drive effective anti-tumor responses in melanoma patients at the cost of toxicity

**DOI:** 10.1007/s00262-016-1796-7

**Published:** 2016-02-10

**Authors:** Kalijn F. Bol, Erik H. J. G. Aarntzen, Jeanette M. Pots, Michel A. M. Olde Nordkamp, Mandy W. M. M. van de Rakt, Nicole M. Scharenborg, Annemiek J. de Boer, Tom G. M. van Oorschot, Sandra A. J. Croockewit, Willeke A. M. Blokx, Wim J. G. Oyen, Otto C. Boerman, Roel D. M. Mus, Michelle M. van Rossum, Chantal A. A. van der Graaf, Cornelis J. A. Punt, Gosse J. Adema, Carl G. Figdor, I. Jolanda M. de Vries, Gerty Schreibelt

**Affiliations:** Department of Tumor Immunology, Radboud Institute for Molecular Life Sciences, Radboud University Medical Centre, PO Box 9101, 6500 HB Nijmegen, The Netherlands; Department of Medical Oncology, Radboud University Medical Centre, Nijmegen, The Netherlands; Department of Hematology, Radboud University Medical Centre, Nijmegen, The Netherlands; Department of Pathology, Radboud University Medical Centre, Nijmegen, The Netherlands; Department of Radiology and Nuclear Medicine, Radboud University Medical Centre, Nijmegen, The Netherlands; Department of Dermatology, Radboud University Medical Centre, Nijmegen, The Netherlands; Department of Pulmonary Diseases, Radboud University Medical Centre, Nijmegen, The Netherlands; Department of Medical Oncology, Academic Medical Centre, Amsterdam, The Netherlands

**Keywords:** Dendritic cells, Immunotherapy, Melanoma, Toll-like receptor ligands, Maturation, Prophylactic vaccines

## Abstract

Dendritic cell (DC)-based immunotherapy is explored worldwide in cancer patients, predominantly with DC matured with pro-inflammatory cytokines and prostaglandin E_2_. We studied the safety and efficacy of vaccination with monocyte-derived DC matured with a cocktail of prophylactic vaccines that contain clinical-grade Toll-like receptor ligands (BCG, Typhim, Act-HIB) and prostaglandin E_2_ (VAC-DC). Stage III and IV melanoma patients were vaccinated via intranodal injection (12 patients) or combined intradermal/intravenous injection (16 patients) with VAC-DC loaded with keyhole limpet hemocyanin (KLH) and mRNA encoding tumor antigens gp100 and tyrosinase. Tumor antigen-specific T cell responses were monitored in blood and skin-test infiltrating-lymphocyte cultures. Almost all patients mounted prophylactic vaccine- or KLH-specific immune responses. Both after intranodal injection and after intradermal/intravenous injection, tumor antigen-specific immune responses were detected, which coincide with longer overall survival in stage IV melanoma patients. VAC-DC induce local and systemic CTC grade 2 and 3 toxicity, which is most likely caused by BCG in the maturation cocktail. The side effects were self-limiting or resolved upon a short period of systemic steroid therapy. We conclude that VAC-DC can induce functional tumor-specific responses. Unfortunately, toxicity observed after vaccination precludes the general application of VAC-DC, since in DC maturated with prophylactic vaccines BCG appears to be essential in the maturation cocktail.

**Electronic supplementary material:**

The online version of this article (doi:10.1007/s00262-016-1796-7) contains supplementary material, which is available to authorized users.

## Introduction

Dendritic cells (DC) have the unique capacity to activate naive tumor-specific T cells [1]. They play a critical role in determining the magnitude and quality of the immune response to an antigen. Immunotherapy applying ex vivo-generated and tumor antigen-loaded DC has now been introduced in the clinic [2, 3]. A limited, but consistent, number of objective immunological and clinical responses have been observed [3]. Thus far, it remains unclear why some patients respond while others do not, but there is a general consensus that the current protocols applied to generate DC may not result in the induction of optimal T helper 1 (Th1) responses and hence cytotoxic T cell responses. We and others have demonstrated that DC maturation is one of the crucial factors to induce effective anti-tumor immune responses in cancer patients [4–7]. Currently, DC are mostly matured with a cocktail of pro-inflammatory cytokines, including IL-1β, IL-6, tumor necrosis factor alpha (TNFα), and prostaglandin E_2_ (PGE_2_). However, DC matured in the presence of Toll-like receptor (TLR) ligands may unleash more potent immune responses, as mouse studies have shown that TLR-matured DC are able to promote T helper 1 cell differentiation and induce full effector T cell differentiation [8]. TLR-mediated maturation of ex vivo-generated human monocyte-derived DC (moDC) may thus be used to improve immunological and clinical responses in DC vaccination of cancer patients.

TLR are pattern recognition receptors that sense microbial and viral products, like bacterial cell wall components or double-stranded RNA. TLR engagement on DC induces maturation and cytokine secretion. In humans, 11 TLR have been described for which many specific ligands have been identified [9, 10]. Whereas several TLR ligands have been shown to yield mature Th1-directing DC, limited availability of Good Manufacturing Practice (GMP)-compliant produced ligands impede the use of these TLR ligands for the generation of DC for immunotherapy in patients. However, prophylactic vaccines against infectious diseases frequently contain molecules derived from bacteria or viruses, which are natural TLR ligands. We identified a cocktail of the clinical-grade prophylactic vaccines BCG, Influvac, and Typhim that contains a multitude of natural TLR ligands and is capable of optimally maturing DC [11]. These so-called prophylactic vaccine-matured DC showed high expression of CD80, CD83, and CD86 and secreted high levels of IL-12. Although these DC exhibited an impaired migratory capacity, this could be restored by addition of PGE_2_. DC matured with prophylactic vaccines and PGE_2_ are potent inducers of T cell proliferation, Th1 polarization, and tumor antigen-specific CD8^+^ effector T cells ex vivo. Prophylactic vaccine-induced DC maturation is compatible with mRNA electroporation as an antigen loading strategy of DC [11]. Here, we studied the safety, immunoreactivity, migratory capacity, and efficacy of intravenous/intradermal (i.v./i.d.) or intranodal (i.n.) vaccination with DC matured with a prophylactic vaccine cocktail, consisting of the clinical-grade prophylactic vaccines BCG, Typhim, and Act-HIB, together with PGE_2_ (VAC-DC) in a dose escalation study in stage III and IV melanoma patients.

## Patients and methods

### Patient population

Melanoma patients with regional lymph node-positive resectable disease (further referred to as stage III), before or within 2 months after radical lymph node dissection, and patients with locally irresectable or distant metastatic disease (further referred to as stage IV) were included. Additional inclusion criteria were melanoma expressing gp100 (compulsory) and tyrosinase (non-compulsory), and WHO performance status 0 or 1. In protocol A HLA-A*02:01 phenotype was an additional inclusion criteria. Patients with brain metastases, serious concomitant disease, use of immunosuppressive drugs, or a history of a second malignancy were excluded. The studies were approved by the Dutch Central Committee on Research involving Human Subjects, written informed consent was obtained from all patients, and all procedures were performed in accordance with the Declaration of Helsinki. ClinicalTrials.gov registration numbers are NCT00940004 (protocol A) and NCT01530698 (protocol B).

### Clinical protocol and immunization schedule

A leukapheresis was performed from which DC were generated. Patients received a VAC-DC vaccine i.v./i.d. (protocol A; 2/3 i.v. and 1/3 i.d.) or i.n. (protocol B; Supplementary Figure 1). Intranodal vaccination was conducted in a clinically tumor-free lymph node under ultrasound guidance. The VAC-DC vaccine consisted of autologous mature moDC electroporated with mRNA coding for gp100 and tyrosinase protein, and pulsed with keyhole limpet hemocyanin (KLH) protein. Patients received three biweekly vaccinations per cycle. Eight patients received an extra vaccination before radical lymph node dissection for additional imaging studies. One to two weeks after the last vaccination, a skin test was performed. In absence of disease recurrence or progression, patients received a maximum of two maintenance cycles at 6-month intervals. All vaccinations were administered between June 2009 and May 2012. Endpoints of this trial were safety, the induction of tumor antigen-specific immune responses, and the clinical response of stage IV patients according to the RECIST1.1 criteria. Toxicity was assessed according to NCI CTC version 3.0.

### DC preparation and characterization

Monocytes were enriched from leukapheresis products by counterflow elutriation using Elutra cell separator (Gambro BCT) and cultured as described [5, 12]. In our preclinical study, we developed a TLR maturation cocktail consisting of BCG, Typhim, and Influvac as clinical-grade alternative for synthetically produced TLR ligands for moDC maturation [11]. Since Influvac is only available during the flu season and has a different composition each year, we replaced Influvac by Act-HIB in our maturation cocktail. Both maturation cocktails gave rise to highly mature, IL-12-producing DC (Supplementary Figure 2). Therefore, in the present study, DC were matured with a cocktail of prophylactic vaccines including BCG vaccine SSI (4 % v/v, Nederlands Vaccin Instituut), Typhim Vi (4 % v/v, Sanofi Pasteur MSD), and Act-HIB (4 % v/v, Aventis Pasteur), supplemented with PGE_2_ (10 µg/ml, Pharmacia and Upjohn) for 48 h (VAC-DC) [11]. For the delayed-type hypersensitivity (DTH) skin test, DC were matured either with the prophylactic vaccine cocktail or with a cytokine cocktail consisting of TNFα (10 ng/ml), IL-1β (5 ng/ml), IL-6 (15 ng/ml) (all CellGenix) and PGE_2_ (10 µg/ml) for 48 h (cDC) [5]. Mature DC were electroporated with GMP-grade gp100 and tyrosinase-encoding mRNA and characterized by flow cytometry as described [13]. The release criteria were: ≥70 % viability, ≥50 % expression of CD83, and expression of MHC class I, MHC class II, CD80, CD86, and CCR7.

### [^111^Indium] labeling and scintigraphy

DC migration was measured after the first vaccination by scintigraphic imaging as described [14]. DC were incubated with ^111^In-oxine (GE Healthcare) in 0.1 ml/l Tris–HCl (pH 7.0) for 15 min at room temperature. Cells were washed three times with PBS, 1 % HSA. In vivo planar scintigraphic images were acquired with a gamma-camera equipped with medium energy collimators, 10 min and 48–72 h after the first vaccination. Migration was quantified by region-of-interest analysis of the individual nodes visualized on the images and expressed as the relative fraction of ^111^In-labeled DC in the injection depot.

### Immunological responses to KLH and prophylactic vaccines

Antibodies against KLH were measured in serum from vaccinated patients by ELISA (www.klhanalysis.com) [15]. Cellular responses against KLH and prophylactic vaccines were measured in a proliferation assay. Peripheral blood mononuclear cells (PBMC) (4 μg/2 × 10^5^) were stimulated with KLH, Act-HIB (4 % v/v), BCG-SSI (4 % v/v), or Typhim Vi (4 % v/v) in medium with 2 % human serum. After 3 days, cells were pulsed with 1 μCi/well tritiated thymidine for 8 h, and incorporation of tritiated thymidine was measured with a beta-counter. A proliferation index >2 was considered positive.

### Proliferative and cytokine response of bronchoalveolar lavage (BAL) cells

Autologous DC of patients V-A-01 and V-A-08 were matured for 48 h with the conventional cytokine cocktail (cDC), the complete prophylactic vaccine cocktail (VAC-DC), or the separate prophylactic vaccines BCG, Typhim, or Act-HIB. cDC was loaded with KLH, gp100 peptides (10 μM gp100:280–288 + 10 μM gp100:154–162), or tyrosinase peptide (10 μM tyrosinase:369–377). 1 × 10^4^ DC were co-cultured with 5 × 10^4^ autologous cells obtained from a bronchoalveolar lavage in RPMI + 7 % human serum. Cytokine production was measured in the supernatant after 24 h by cytometric bead array (human Th1/Th2 11 plex kit, eBioscience) or standard sandwich ELISA (human IL-17 DuoSet ELISA, R&D Systems). To study T cell proliferation, cells were pulsed after 4 days with 1 μCi/well tritiated thymidine for 8 h, and incorporation of tritiated thymidine was measured with a beta-counter.

### MHC tetramer staining

SKIL and PBMC were stained with tetrameric MHC complexes containing HLA-A*02:01 epitopes gp100:154–162, gp100:280–288, or tyrosinase:369–377 (Sanquin). HIV tetramers were used as a negative control.

### Skin-test infiltrating-lymphocytes cultures

One to two weeks after the last DC vaccination, a DTH skin test was performed, as described (https://www.labtube.tv/video/Skin-test-infiltrating-lymphocyte-SKIL-test-120284) [16, 17]. For HLA-A*02:01-positive patients, antigen recognition was determined by the production of cytokines of SKIL after co-culture with T2 cells pulsed with the indicated peptides or BLM (a melanoma cell line expressing HLA-A*02:01 but no endogenous expression of gp100 and tyrosinase), transfected with control antigen G250, gp100 or tyrosinase, or an allogeneic HLA-A*02:01-, gp100-, and tyrosinase-positive tumor cell line (MEL624). Cytokine production was measured in supernatants after 24 h of co-culture with a FlowCytomix Multiplex kit (Bender MedSystems GmbH). For HLA-A*02:01-negative patients, antigen recognition by SKIL was determined using autologous EBV-transformed B (EBV-B) cells electroporated with gp100-mRNA or tyrosinase mRNA as described [18, 19].

### Statistical analysis

Planned patient accrual was 25 in protocol A and 17 in protocol B. Data were analyzed statistically by means of analysis of variance and Student–Newman–Keuls test, or by means of Mann–Whitney U nonparametric statistics. Statistical significance was defined as *p* < 0.05. Progression-free and overall survival were calculated from the time from apheresis to disease recurrence (for stage III patients) or progression (for stage IV patients) or death. Differences between Kaplan–Meier estimates of survival times were assessed using the log-rank test.

## Results

### Patient characteristics and vaccination cycles

A total of 29 melanoma patients, 11 stage III patients and 18 stage IV patients, were included. One stage IV melanoma patient showed rapid progressive disease with signs of spinal cord compression before vaccination started and went off study to receive local treatment. Sixteen patients were vaccinated i.v./i.d (protocol A); 12 patients were vaccinated i.n. (protocol B; Supplementary Figure 1). Patient characteristics are summarized in Table 1.

**Table 1:** Patient characteristics

	Patient	Sex	Age	N or M stage^a^	Baseline LDH	Site of disease	Number of metastasis	Gp100^b^	Tyrosinase^b^	HLA-A*02:01 status	Mutation status	Post-DC treatment
		F/M	Yrs		U/l			Intensity	Intensity			
*i.v./i.d.*					ULN < 450							
Stage IV	A-1	F	50	M1c	663	Liver, lung, skin	>10	+	−	+	wt	S
	A-2	F	66	M1a	383	Distant LN	>5	+++	+++	+	wt	−
	A-3	M	60	M1b	396	Distant LN, lung	>5	pos	−	+	wt	S, C, I
	A-4	M	65	M1b	368	Lung	4	+++	−	+	wt	C
	A-5	M	32	M1c	329	Liver, distant LN, soft tissue	>5	pos	n.t.	+	n.t.	S
	A-6	M	37	M1c	389	Liver, lung, bone, skin, cardiac	>10	+++	+++	+	n.t.	−
	A-7	M	53	M1c	517	Liver, bone	>5	+	−	+	n.t.	I
	A-8	M	55	N3irr	445	Inguinal + paraaortic LN	>5	+++	+	+	n.t.	−
	A-9	F	35	M1a	269	Skin	2	+++	+++	+	BRAF	S
Stage III	A-10	M	46	N2b	340	Cervical LN	2	+++	+++	+	BRAF	S, T1, I
	A-11	F	51	N1b	431	Inguinal LN	1	+++	++	+	n.t.	n.a.
	A-12	M	60	N1b	372	Axillary LN	1	+++	+	−	n.t.	n.a.
	A-13	M	64	N3	287	Cervical LN	5	++	++	−	NRAS	T2
	A-14	F	43	N3	385	Cervical LN	>5	+++	+++	+	BRAF	−
	A-15	M	51	N3	421	Inguinal LN	>10	++	++	+	n.t.	S
	A-16	M	53	N2b	337	Inguinal LN	2	+++	+++	+	NRAS	−
*i.n.*												
Stage IV	B-1	M	60	M1b	427	Distant LN, lung, skin	>5	+++	+++	+	BRAF	S, I, T1
	B-2	M	48	M1b	321	Lung, skin	5	++	−	−	n.t.	I
	B-3	M	42	M1a	450	Distant LN, skin	>10	+++	+++	+	wt	I, S
	B-4	M	69	M1b	381	Distant LN, lung	>5	++	++	+	BRAF	C, T1
	B-5	M	57	M1c	251	Bone	1	+++	++	−	n.t.	C
	B-6	M	29	N3 irr	341	Axillary LN + in transit mets	>10	+++	+	−	NRAS	C, I, T2
	B-7	M	63	M1b	340	Lung	2	++	++	−	wt	S, I
	B-8	F	56	M1a	267	Distant LN, skin	5	+++	+++	−	BRAF	−
Stage III	B-9	F	57	N3	312	Inguinal LN	>5	+++	++	+	wt	−
	B-10	M	72	N3	353	Cervical LN	>5	+++	++	−	n.t.	n.a.
	B-11	M	37	N3	296	Inguinal LN	4	+++	+++	−	BRAF	T1
	B-12	M	26	N2a	353	Axillary LN	2	+++	+++	−	n.t.	n.a.

*BRAF* BRAF mutation present, *C* chemotherapy, *I* immunotherapy (anti-CTLA-4), *S* surgery, *n.a.* not applicable, *NRAS* NRAS mutation present, *n.t.* not tested, *T1* targeted therapy (BRAF inhibitor), *T2* targeted therapy (MEK inhibitor), *wt* wild type (no BRAF or NRAS mutation present)

^a^As per pathology report of the radical lymph node dissection in stage III melanoma patients and per CT scan in stage IV melanoma patients

^b^gp100 and tyrosinase expression on the primary tumor was analyzed by immunohistochemistry. Intensity of positive cells was scored centrally and semi-quantitatively by a pathologist. Intensity was scored as low (+), intermediate (++), or high (+++), or not scored (pos)

In the i.v./i.d. group, the first five patients received increasing doses of VAC-DC (7.5 to 30 × 10^6^ DC). Eight additional patients received the full dose of maximally 30 × 10^6^ VAC-DC. Due to serious side effects (see below), the maximum dose was reduced to 15 × 10^6^ DC. As toxicity did not diminish after dose reduction, the inclusion of patients in protocol A was terminated. In the i.n. group, the first five patients received increasing doses of VAC-DC (1.5–15 × 10^6^ DC). An additional seven patients received the maximum dose of 15 × 10^6^ VAC-DC. Due to serious side effects (see below), none of the patients in the i.n. group received maintenance cycles, and further inclusion of patients was stopped.

### Characteristics of injected DC

After maturation, VAC-DC of all patients had a mature phenotype based on expression of MHC class I and II, co-stimulatory molecules, CD83, and CCR7 (Supplementary Figure 3a). Intracellular expression of tumor-associated antigens gp100 and tyrosinase after electroporation was variable, but for each patient expression of either gp100 or tyrosinase was at least 30 % (Supplementary Figure 3b).

### Migratory capacity of VAC-DC

**Fig. 1:**
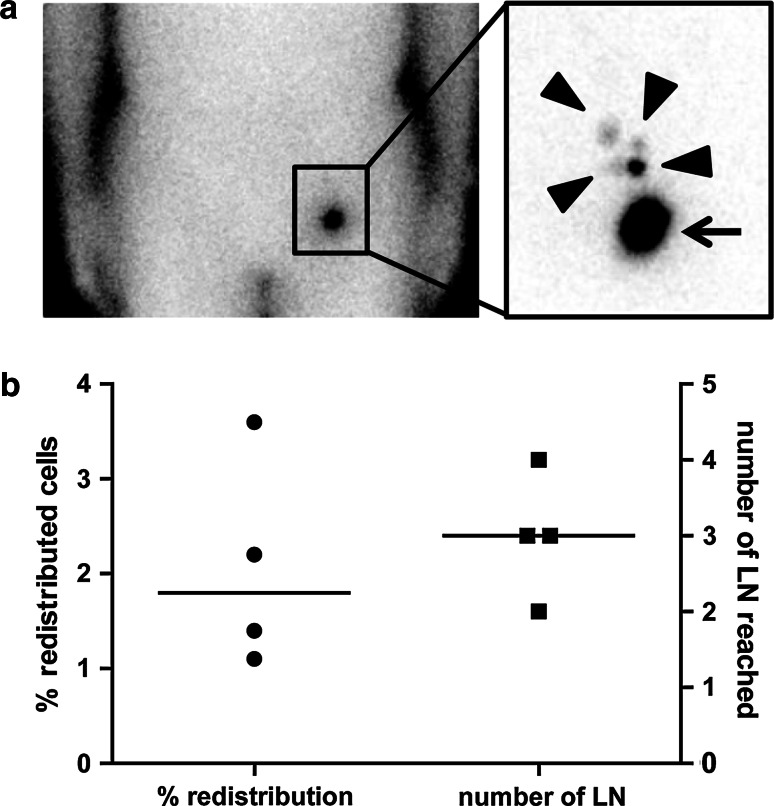
VAC-DC migration after intradermal injection. In four patients VAC-DC migration to nearby lymph nodes (LN) was analyzed by scintigraphy of the lymph node region 48–72 h after intradermal injection of ^111^Indium-labeled VAC-DC. **a** Example of a scintigraphic image showing the redistribution to multiple lymph nodes of ^111^Indium-labeled DC from the injection depot (*arrow*) to four nearby LN (*arrow heads*) in patient A-13. **b** Percentage of cells migrated to nearby LN (*left*) and number of reached LN (*right*). One *symbol* represents a single patient who received maximally 10 × 10^6^ cells by intradermal injection; *horizontal lines* represent the median

In four patients the distribution of ^111^Indium-labeled VAC-DC was determined by scintigraphic imaging 10 min and 2–3 days after the first i.d. vaccination. In all four patients, VAC-DC migrated from the injection depot to multiple nearby lymph nodes (Fig. 1a). The median overall redistribution of injected DC was 1.8 % (range 1.1–3.6 %), and median 3 lymph nodes were reached (range 2–4; Fig. 1b). These results demonstrate that VAC-DC have the capacity to migrate towards lymph nodes after i.d. injection.

### Flu-like symptoms and injection site reactions

Almost all patients vaccinated with VAC-DC experienced CTC grade 2 toxicity with higher fever and stronger injection site reaction as compared to patients vaccinated with cytokine-matured DC (cDC) in previous studies (Table 2, Supplementary Figure 4a). Interestingly, two patients in the i.v./i.d. group (A-2 and A-3) showed re-appearance of induration of the injection site of recent i.d. VAC-DC vaccination after regular seasonal flu vaccination (Supplementary Figure 4b), whereas the flu vaccine was not part of the maturation cocktail. In the i.n. group, the injection site reactions induced substantial lymphadenopathy and erythema of the overlying skin. In some cases it was accompanied by purulent discharge, resembling suppurative lymphadenitis (Supplementary Figure 4c+d).

**Table 2:** Immunological and clinical responses

	Patient	Cycles of VAC-DC	Flu-like symptoms (CTC grade)	Injection site reaction (CTC grade)	Hepatotoxicity (CTC grade)	Pneumonitis	Recurrence free (st. III) or progression-free (st. IV) survival^k^ (months)	Overall survival (months)	Best clinical response	Tetramer-positive CD8 + T cells in blood^c^	Tumor antigen-specific T cells in SKIL cultures			
											CD8 + Tetramer +^c^	Peptide^d^	Protein^d^	Tumor^d^
*i.v./i.d.*														
Stage IV	A-1	1	1	2	2	No	2	7	PD	−	−	n.t.	n.t.	n.t.
	A-2	1	2	2	1	No	4	21	PD	+	+	−	−	−
	A-3	2	1	1	2	No	9^b^	19	SD	+	+++	−	−	−
	A-4	1	1	2	2	No	2	11	PD	−	−	−	−	−
	A-5	1	2	1	2	No	2	10	PD	+	−	−	−	−
	A-6	1	2	0	1	Possible	2	3	PD	−	−	−	−	−
	A-7	1	1	0	3	No	2	4	PD	−	−	−	−	−
	A-8	1	1	1	2	Possible	1	10	PD	−	−	−	−	−
	A-9	1	2	1	3	Yes	1	54+	PD	−	n.a.	n.a.	n.a.	n.a.
Stage III	A-10	3^a^	2	1	3	Yes	50^b^	64+	NED	−	+++	+	+	−
	A-11	2^a^	2	2	3	Yes	59+	59+	NED	−	++	−	−	−
	A-12	3	2	1	1	No	53+	53+	NED	n.a.	n.a.	n.a.	++	+
	A-13	2^a^	3	2	3	Yes	10	18	NED	n.a.	n.a.	n.a.	−	−
	A-14	1	1	1	2	No	7	7	NED	−	−	−	−	−
	A-15	3	1	1	1	No	36	46+	NED	+	++	+++	++	+
	A-16	2	2	1	2	No	10	12	NED	++	++	−	−	−
*i.n.*														
Stage IV	B-1	1	1	1	1	No	2	29	PD	++	+++	+	−	−
	B-2	1	2	0	2	No	6	10	SD	n.a.	n.a.	n.a	n.t.	n.a
	B-3	1	2	2	1	No	4	8	SD/MR	−	−	−	−	−
	B-4	1	1	2	1	No	2	14	PD	+	−	−	−	−
	B-5	1	2	1	2	No	2	12	PD	n.a.	n.a.	n.a	−	n.a
	B-6	1	1	2	0	No	2	14	PD	n.a.	n.a.	n.a	−	n.a
	B-7	1	1	2	1	No	2	36	PD	n.a.	n.a.	n.a	−	n.a
	B-8	1	2	2	1	No	8	14	SD	n.a.	n.a.	n.a	−	n.a
Stage III	B-9	1	2	2	0	No	19	22	NED	−	++	−	+	+
	B-10	1	2	1	0	No	51+	51+	NED	n.a.	n.a.	n.a	−	n.a
	B-11	1 a	2	2	0	No	28	42	NED	n.a.	n.a.	n.a	++	n.a
	B-12	1	2	2	2	No	47+	47+	NED	n.a.	n.a.	n.a	−	n.a

*n.a.* not applicable, *n.t.* not tested, *PD* progressive disease, *SD* stable disease, *NED* no evidence of disease, *MR* mixed response, *SKIL* skin-infiltrating lymphocytes

^a^Cycle was stopped due to adverse events

^b^Previous local relapse resected

^c^Tetramer staining of freshly isolated peripheral blood mononuclear cells or SKIL. −, no recognition; +, 1 epitope recognized; ++, 2 epitopes recognized; +++, 3 epitopes recognized

^d^For HLA-A*02:01-positive patients, antigen recognition by SKIL was analyzed by stimulation of SKIL with T2 cells loaded with HLA-A2.1-binding gp100 or tyrosinase peptides (peptide recognition), BLM transfected with gp100 or tyrosinase protein (protein recognition) or the gp100- and tyrosinase-expressing tumor cell line Mel624 (tumor recognition) as analyzed by IFNγ production. For HLA-A*02:01-negative patients, antigen recognition by SKIL was analyzed by stimulation of SKIL with autologous PBL or EBV-transformed B cells electroporated with gp100 or tyrosinase mRNA, as analyzed by expression of either CD69, CD137, or CD107a or production of IFNγ. Responses were scored as the best immunologic response after 1 to 3 cycles of DC vaccinations. −, no recognition; +, 1 epitope/antigen recognized; ++, 2 epitopes/antigens recognized; +++, 3 epitopes recognized

Remarkably, patient A-3 showed vitiligo on the chest and back after the second cycle of i.v./i.d. VAC-DC vaccinations. The occurrence of vitiligo in patients with melanoma is reported for patients undergoing immunotherapy and can be an indication of an immune response directed against melanoma/pigmented cells and correlate with survival [20, 21].

### Hepatotoxicity and pneumonitis

In all but four patients hepatotoxicity was observed. A rise in liver enzymes was most pronounced after i.v./i.d. injection and occurred in five patients up to CTC grade 3 severity (Table 2). Although in some patients progressive liver metastases could not be excluded as a causative factor, the transient increase of liver tests during the vaccination cycle in most patients clearly support a toxic effect of VAC-DC vaccination. The increases in liver tests returned to baseline within 1 month after vaccination and were not accompanied by alterations in bilirubin.

**Fig. 2:**
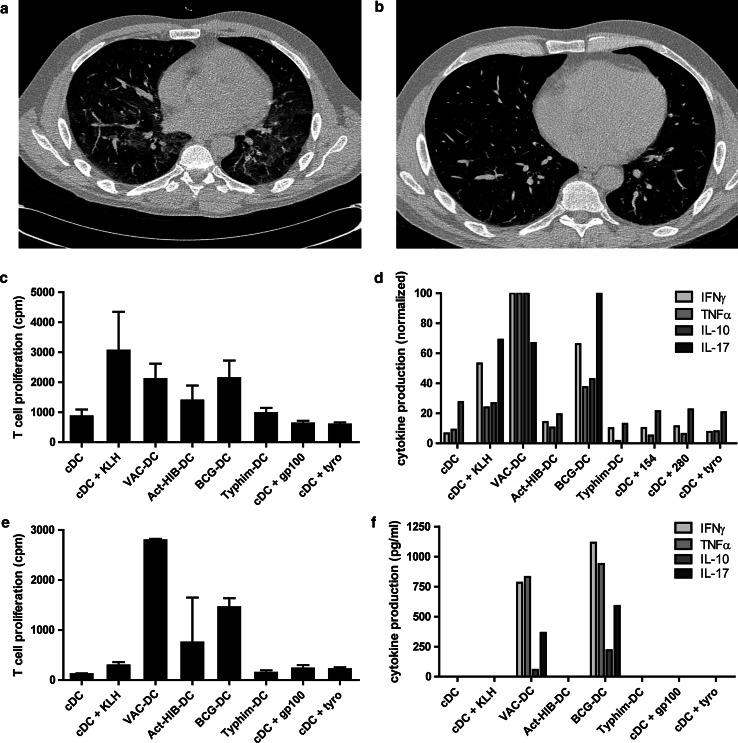
VAC-DC-induced lung toxicity. Example of high-resolution CT scan (patient A-10) showing diffuse infiltration in the lungs suggestive of pneumonitis (**a**), which resolved after short treatment with systemic steroids (**b**). Cells obtained from bronchoalveolar lavage of patients A-9 (**c**, **d**) and A-10 (**e**, **f**) were co-cultured with autologous DC loaded with KLH, gp100, tyrosinase, the prophylactic vaccine cocktail, or with BCG, Typhim, or Act-HIB. **c**, **e** T cell proliferation was measured in triplicate by incorporation of tritiated thymidine after 4 days. **d**, **f** Cytokine production was measured in the supernatant after 24 h by cytometric bead array and ELISA. In **f**, cytokine production is normalized to the highest value, due to large differences in concentration between the different cytokines. Maximum cytokine concentrations (100 %) were: IFNγ 9.7 ng/ml; TNFα 328 ng/ml; IL-10 161 ng/ml; IL-17 181 pg/ml. In conclusion, cells obtained from the bronchoalveolar lavage of both patients showed that infiltrated cells were BCG specific; this might have caused the development of pneumonitis

Six patients in the i.v./i.d. group presented with acute onset of dyspnea and dry cough. In the first two patients a CT-angiography scan was made of which the results excluded a pulmonary embolism. High-resolution CT scans of these patients and two others showed diffuse increased density of the lung parenchyma, classified as interstitial pneumonitis (Fig. 2a). All four patients were treated with a short course of systemic steroids, resulting in improvement in dyspnea within 2 days. The CT abnormalities resolved in one to 3 months (Fig. 2b). Two other patients presented with similar symptoms but did not show signs of pneumonitis on a planned CT scan for response evaluation. A high-resolution CT scan was not performed in these patients. A planned CT scan showed a segmental pulmonary embolism in one patient, which was considered to be a coincidental finding as this patient had no pulmonary complaints at that time.

Immune cells obtained from a BAL of patients A-9 and A-10 proliferated and produced interferon gamma (IFNγ) and TNFα when co-cultured with autologous VAC-DC or BCG alone. BAL-derived immune cells of patient A-9 also responded to KLH, but did not proliferate upon stimulation with gp100 or tyrosinase peptides. In addition, staining with tetrameric MHC complexes could not demonstrate the presence of tumor antigen-specific T cells in the BAL fluid. These data suggest that at least part of the infiltrated cells were BCG-specific (Fig. 2c–f).

### KLH- and BCG-specific immune responses

**Fig. 3:**
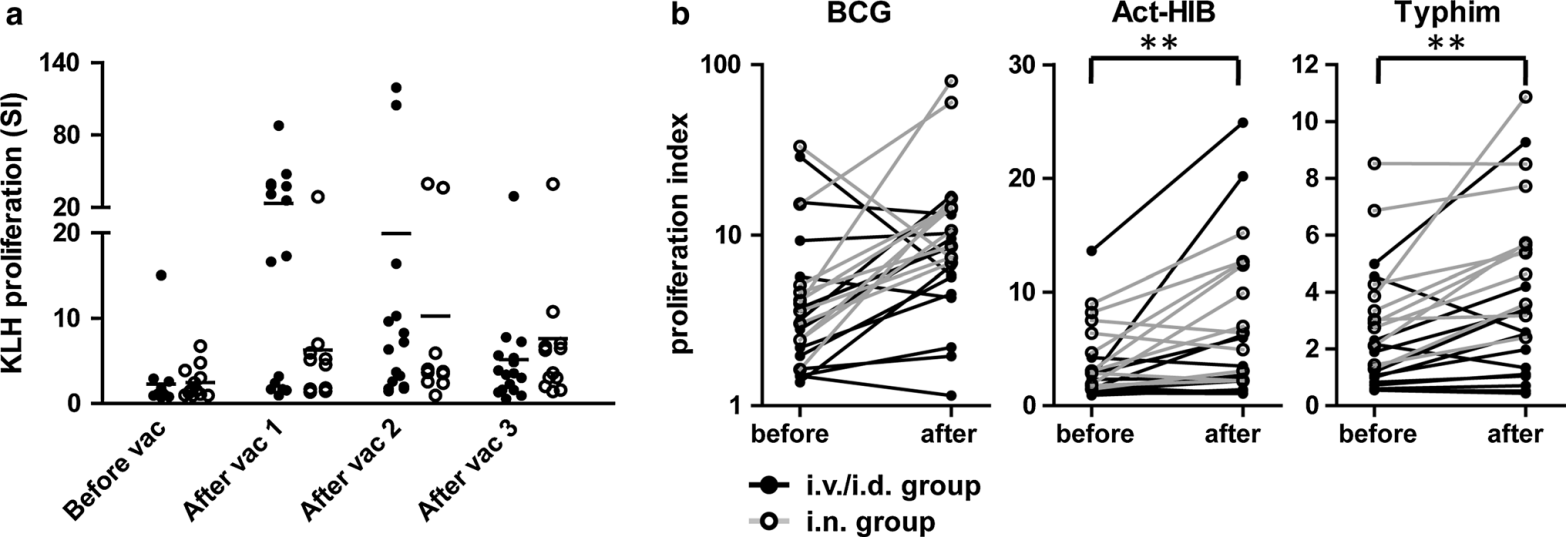
KLH- and prophylactic vaccine-specific T cell responses before and after VAC-DC vaccination. **a** KLH-specific T cell proliferation was analyzed before the first vaccination and after each VAC-DC vaccination during the first vaccination cycle in PBMC. Per time point *each dots* represents one patient; *black dots* represent patients that received i.v./i.d. VAC-DC vaccination, *open dots* represent patients that received i.n. VAC-DC vaccination. *Horizontal lines* represent group averages per time point. In all patients except one, a KLH-specific T cell response was induced. **b** BCG-, Act-HIB-, and Typhim-specific T cell proliferation was analyzed before and after VAC-DC vaccination in PBMC. Proliferative responses to KLH or prophylactic vaccines are given as proliferation index (proliferation with KLH or vaccines/proliferation without KLH or vaccines). **p < 0.01, paired *t* test

To test the capacity of the patients in this study to generate an immune response, we loaded the VAC-DC with the control antigen KLH. All 16 evaluable patients in the i.v./i.d. group and 11 out of 12 patients in the i.n. group showed increased T cell proliferation upon stimulation with KLH, irrespective of the dose of DC administered (Fig. 3a). The only patient who did not show an increased T cell response after i.n. vaccination with VAC-DC already had a T cell response and KLH-specific antibodies in serum before vaccination. Overall, these data demonstrate that both i.v./i.d. injected VAC-DC and i.n. injected VAC-DC effectively induced de novo immune responses in melanoma patients.

For some patients, we also analyzed the induction of T cells specific for the prophylactic vaccines that were used for VAC-DC maturation. As expected, prophylactic vaccine-specific T cell responses were already present before VAC-DC vaccination in some patients (Fig. 3b). However, for all three prophylactic vaccines, increased T cell responses were found in a part of the patients, indicating that the prophylactic vaccines that are used for VAC-DC maturation are processed by the DC and presented to specific T cells after injection.

### Tumor antigen-specific T cell responses

**Fig. 4:**
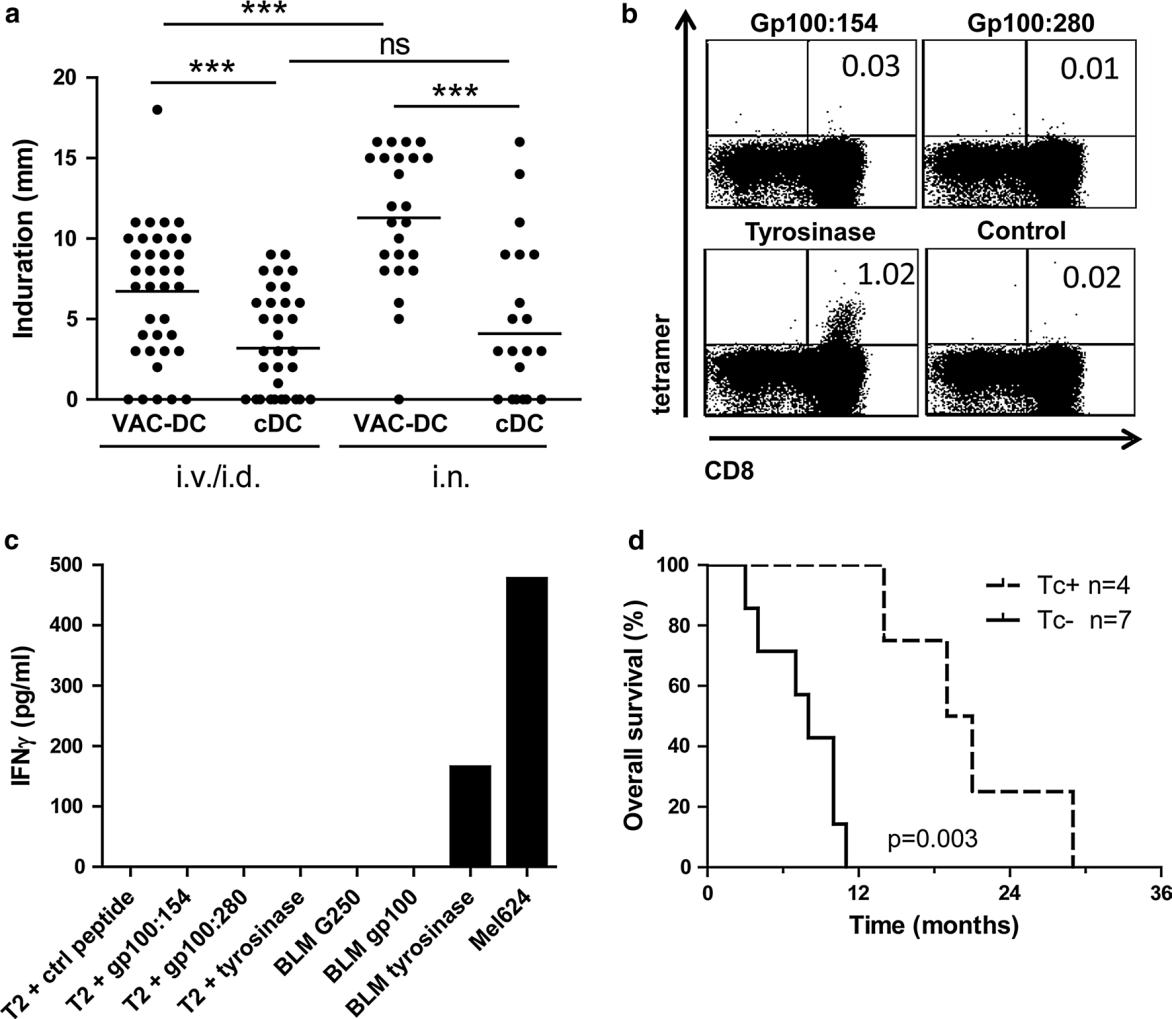
Tumor antigen-specific T cell responses in skin-test infiltrating-lymphocyte cultures. **a** Induration of delayed-type hypersensitivity (DTH) sites measured 48 h after intradermal injection of VAC-DC or cytokine-matured DC (cDC) loaded with gp100 mRNA or tyrosinase mRNA. Data are shown in mm induration. *Each dot* represents one DTH site. The *line* indicates the mean of DTH sites. *** *p* < 0.001, *ns* not significant, paired *t* test. **b** Example of tetramer staining of T cells cultured from a DTH site of patient B-9. Cells were stained with allophycocyanin-labeled tetramers encompassing the gp100:154 peptide, gp100:280 peptide, tyrosinase peptide, or control peptide and with CD8-FITC. *Numbers* indicate the percentage of tetramer-positive cells CD8^+^ T cells of total CD8^+^ T cells. **c** IFNγ production by the same T cells of patient B-9 after stimulation with T2 cells loaded with tumor peptides or BLM cells expressing tumor proteins. **d** Kaplan–Meier analyses of overall survival according to the presence of tetramer-positive populations in skin-test infiltrating-lymphocyte cultures from DTH skin-test biopsies or in peripheral blood in HLA-A*02:01-positive stage IV patients. The presence of tumor antigen-specific T cells (Tc+) correlates with longer overall survival after VAC-DC vaccination in metastatic melanoma patients compared to patients without detectable tumor antigen-specific T cells (Tc-)

To study tumor antigen-specific T cell responses, DTH skin tests were performed. Induration of sites injected with VAC-DC was significantly stronger than at sites injected with cDC (Fig. 4a).

To investigate whether tumor-associated antigen (TAA)-specific immune responses were induced by vaccination with VAC-DC, PBMC and SKIL of HLA-A*02:01-positive patients were screened with tetrameric MHC complexes. After i.v./i.d. vaccination, tetramer-positive PBMC were detected in peripheral blood of three out of nine stage IV patients and two out of five stage III patients tested. Tetramer-positive SKIL were detected in two out of eight stage IV patients tested and four out of five stage III patients tested (Table 2). In none of the i.v./i.d. vaccinated stage IV patients, SKIL produced cytokines upon co-culture with peptide- or protein-loaded target cells, whereas in three stage III patients SKIL recognized endogenously processed tumor proteins. After i.n. vaccination, tetramer-positive CD8^+^ T cells were detected in peripheral blood of two out of three stage IV patients, whereas tetramer-positive SKIL were detected in two out of four HLA-A*02:01-positive patients, one stage IV, and one stage III patient. Interestingly, SKIL of patient B-9 produced IFNγ upon co-culture with tumor protein and not with HLA-A*02:01-binding peptides (Fig. 4b, c), indicating that T cells recognized different epitopes.

In one HLA-A*02:01-negative stage III patients (patient B-11), SKIL produced IFNγ upon co-culture with EBV-B cells, but without concomitant upregulation of CD69 or CD107a. Nevertheless, our analysis for HLA-A*02:01 presented epitopes demonstrate that VAC-DC can induce or enhance tumor-specific immune responses in melanoma patients both after i.v./i.d. and i.n. injection.

### Clinical outcome in stage III patients

The median follow-up was 46 months (range 7–64). In the i.v./i.d. group, five patients had recurrence of disease, of whom three patients died and two patients are alive with disease. In the i.n. group, two patients had recurrence of disease and died. In both groups, two patients have no evidence of disease (Table 2).

### Clinical outcome in stage IV patients

The median follow-up was 12 months (range 3–54). All stage IV patients were evaluated for clinical response at 3-month intervals with CT scans or at earlier time points when progressive disease was clinically suspected. In the i.v./i.d. group, one patient had stable disease and received a second vaccination cycle. The remaining eight patients showed progressive disease prior to or at first evaluation. Patient A-9 is still alive with follow-up of nearly 4 years (April 2015), and the other eight patients died between 3 and 21 months. Mutation status and subsequent treatments after progressive disease are shown in Table 1.

In the i.n. group, three patients had stable disease (8–14 months), of which one had a mixed response, showing a reduction in size of mediastinal lymph node metastasis and a increase in size of abdominal lymph node metastasis. The other five patients showed progressive disease at the first clinical evaluation. All patients died between 8 and 36 months (Table 2).

Despite the small sample size, our data suggest a correlation between the immunological responses and survival of HLA-A*02:01-positive patients, with an overall survival ranging from 14 to 28 months in patients with TAA-specific T cells (*n* = 4), whereas in the absence of these cells (*n* = 7) the overall survival ranges from 3 to 11 months (*p* = 0.003; Fig. 4d).

## Discussion

Based on our in vitro data, showing the potential of DC matured by a cocktail of three prophylactic vaccines (BCG, Typhim, and Influvac or Act-HIB) and PGE_2_ [11], we initiated a study on the safety and the capacity to induce immune responses against tumor antigens of VAC-DC in vivo. Our major conclusions are (1) VAC-DC can induce tumor antigen-specific T cell responses, both after i.v./i.d. and i.n. injection; (2) VAC-DC induce more severe side effects as compared to cDC matured with a conventional cytokine cocktail.

Side effects of cDC vaccines are usually mild and if present include low-grade flu-like symptoms and local reaction at the injection site. Compared with our experience with cDC vaccination [4, 13, 22, 23] and the experience of other groups with Trimix-matured or α type 1-polarized moDC [7, 24, 25], in the present study with VAC-DC vaccination side effects were of higher grade and occurred more often as well as earlier after the first vaccination. Injection site reactions are uncommon upon i.n. injection with cDC [26]; however, upon VAC-DC vaccination substantial lymphadenopathy and erythema of the overlying skin were observed with purulent discharge occurring in some patients. In addition, flu-like symptoms were more severe after VAC-DC vaccination compared to cDC vaccination and were more often accompanied by the presence of fever. Both the injection site reactions and flu-like symptoms were self-limiting but dissolved less rapidly. The foreign KLH antigen in cDC vaccines is regarded a major cause of fever and flu-like symptoms after vaccination with cDC. However, in VAC-DC the BCG vaccine may be responsible for both the side effects, since prophylactic BCG vaccination, intravesicular BCG treatment in bladder cancer patients, and active specific immunotherapy with BCG in colon carcinoma patients are known to induce flu-like symptoms, fever and suppurative lymphadenitis, resembling the clinical picture we observed, as well as pulmonary infiltrates and increased liver function tests [27–29]. The relation between the pneumonitis and the usage of the BCG vaccine in the maturation cocktail of the VAC-DC was substantiated by the observed proliferation and cytokines production in immune cells obtained from BAL in response to stimulation with BCG antigens. We hypothesize that VAC-DC trapped in the lungs after i.v. injection attract BCG-specific immune cells, thereby causing pneumonitis. Symptoms started in the second cycle of vaccinations in three patients and in the first cycle (after the second vaccination) in one patient with a proven pneumonitis. This suggests that the BCG-specific cells were induced by the first round of VAC-DC vaccinations. The patient who developed pneumonitis after the second vaccination had a very high BCG-specific T cell proliferation index before vaccination, suggesting that these cells were already present prior to VAC-DC vaccination. Attempts to replace or remove BCG from the maturation cocktail consisting of prophylactic vaccines have so far been unsuccessful, as BCG appears to be essential to obtain IL-12-producing DC with a mature phenotype [11]. Both protocols were prematurely terminated; this was mainly due to the pulmonary toxicity (protocol A) that occurred and the extensive injection site reactions (protocol B).

For DC to induce an effective immune response, it is crucial to migrate to the T cell areas of the lymph node after injection. VAC-DC express CCR7, and our in vivo data show that after i.d. injection VAC-DC migrate towards regional lymph nodes. Compared to our previous migration studies with i.d. injected cDC [30], i.d. injected VAC-DC migrate in a comparable percentage to nearby lymph nodes but to a somewhat larger number of nodes. Our in vitro studies showed that addition of PGE_2_ to the vaccine cocktail is needed to obtain DC that are responsive to lymph node chemokines [11]. However, PGE2 also has suppressive activities, including suppression of IL-12 production by DC [31]. Indeed, in our in vitro studies, addition of PGE2 to the maturation cocktail reduced IL-12 production. However, secreted IL-12 levels were still 100-fold higher than levels secreted by cytokine-matured DC and sufficient to induce IFNγ-producing Th1 cells [11].

Vaccination with VAC-DC induced tumor antigen-specific CD8^+^ T cell responses, both after i.v./i.d. injection and after i.n. injection. Previously, we showed that the presence of tumor antigen-specific T cells in DTH skin tests positively correlates with clinical outcome in metastatic melanoma patients after cDC vaccination [16, 17]. Although groups are too small to draw firm conclusions, we found a similar correlation between the immunological responses and overall survival in stage IV HLA-A*02:01-positive melanoma patients. In line with our previous studies [13, 16, 32], robust immunologic responses were more frequently detected in patients with no evidence of disease (stage III melanoma) than in patients with macroscopic tumor burden (stage IV). This is in line with the hypothesis that high tumor burden may hamper the induction of effective immune responses by the secretion of suppressive cytokines and attraction of regulatory T cells [33].

In the i.v./i.d. group the percentage of patients with immunological responses was comparable to that after vaccination with cDC [16]. By contrast, in the i.n. group very few tumor antigen-specific immune responses were detected. The majority of patients in this group were HLA-A*02:01 negative, which is, however, not a prognostic factor in melanoma but might be predictive for response to DC vaccination [34]. In theory, as DC were loaded with mRNA encoding the whole TAA, this obviates HLA restriction and allows immune responses against a broad array of epitopes. Unfortunately, the monitoring of tumor antigen-specific immunological responses in HLA-A*02:01-negative patients is far more complicated, and the alternative approach using EBV-B cells might not allow the detection of all tumor antigen-specific immune responses [18]. Furthermore, the low frequency of tumor antigen-specific immune responses may be due to the i.n. injection route [35], which may cause partial destruction of the lymph node architecture. Additionally, after i.d. injection, the DC that reach the lymph nodes may represent the most mature and hence most potent DC. Lastly, patients in the i.n. group received lower number of vaccinations, due to treatment discontinuation for reasons of toxicity.

We conclude that vaccination of melanoma patients with VAC-DC results in functional tumor antigen-specific T cell responses, however, at the cost of substantial toxicity. This impedes the general application of VAC-DC, as DC-based immunotherapy nowadays competes with the immune checkpoint inhibitors, and its main advantages are the limited toxicity and maintenance of good quality of life. To avoid this toxicity but still allow the benefit from VAC-DC-induced immunological responses, GMP-grade purified TLR ligands may be an alternative.

## Electronic supplementary material

Supplementary material 1 (PDF 346 kb)

## Abbreviations

BAL: Bronchoalveolar lavage
cDC: Cytokine-matured DC
DC: Dendritic cell(s)
DTH: Delayed-type hypersensitivity
GMP: Good manufacturing practice
i.d.: Intradermal
IFNγ: Interferon gamma
i.n.: Intranodal
i.v.: Intravenous
KLH: Keyhole limpet hemocyanin
moDC: Monocyte-derived DC
PBMC: Peripheral blood mononuclear cells
PGE_2_: Prostaglandin E_2_
SKIL: Skin-test infiltrating-lymphocytes
TAA: Tumor-associated antigen(s)
Th1: T helper 1
TLR: Toll-like receptor(s)
TNFα: Tumor necrosis factor alpha
VAC-DC: DC matured with prophylactic vaccines and PGE_2_
