# Supplementary material for: Prophylactic vaccines are potent activators of monocyte-derived dendritic cells and drive effective anti-tumor responses in melanoma patients at the cost of toxicity
Source: Cancer Immunol Immunother. 2016 Feb 10;65(3):327–39. doi: 10.1007/s00262-016-1796-7 (PMC4779136; doi:10.1007/s00262-016-1796-7)
Supplement: Supplementary file 1 — Supplementary material 1 (PDF 346 kb) [file 262_2016_1796_MOESM1_ESM.pdf]

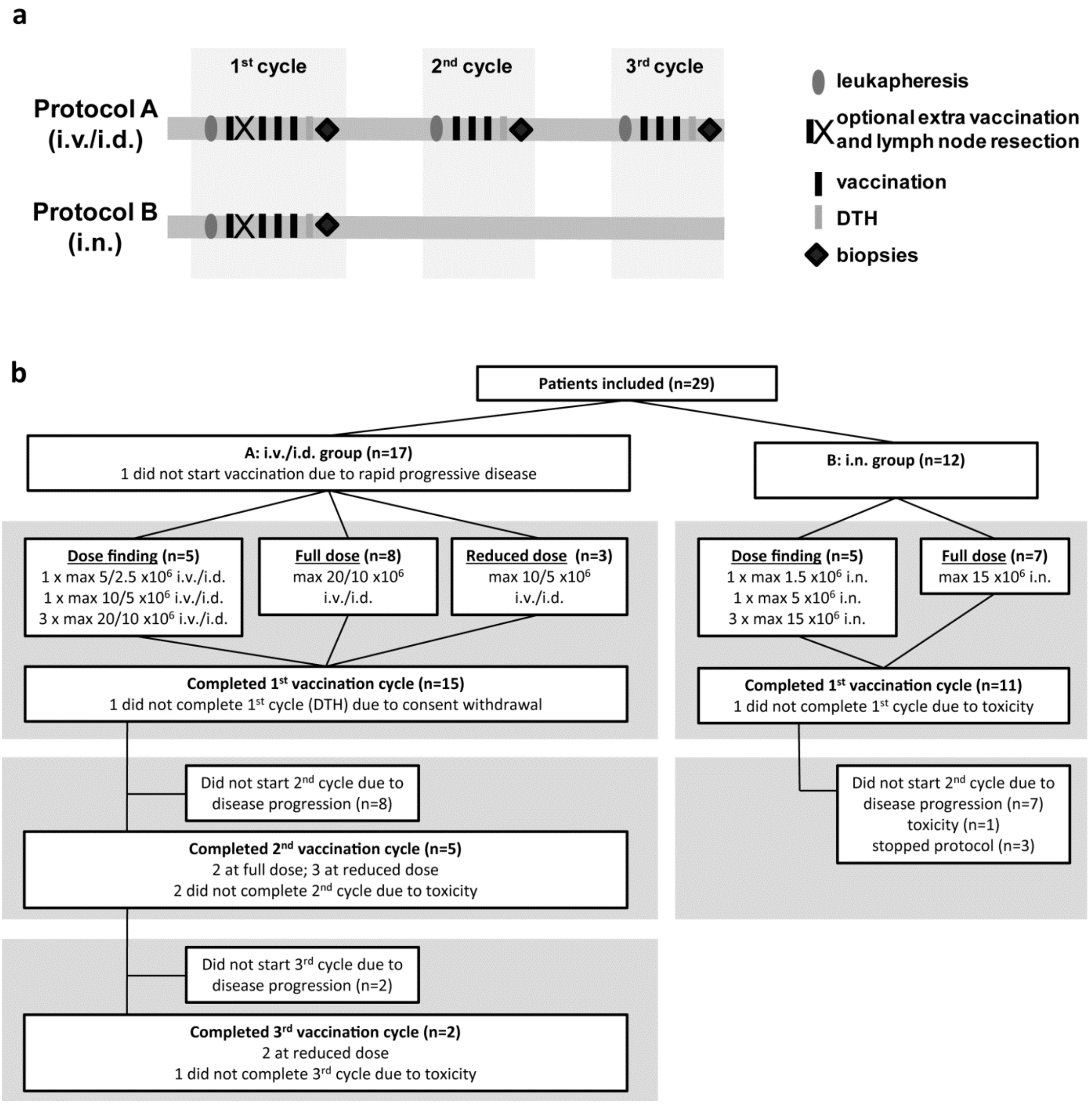

Supplementary Figure 1. Schematic treatment schedule and CONSORT flow chart

(**a**) Schematic treatment schedule. (**b**) CONSORT flow chart.

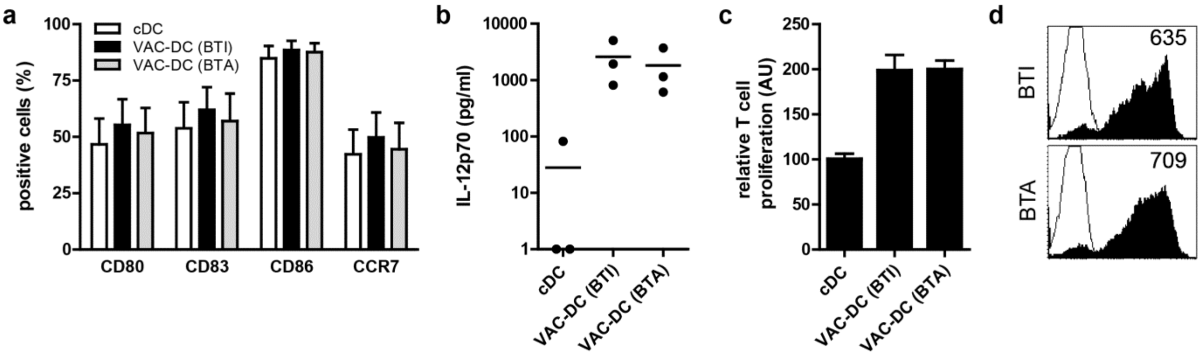

Supplementary Figure 2. Prophylactic vaccine maturation cocktail with Influvac compared to with Act-HIB

Monocyte-derived DC were matured for 48h with the conventional cytokine cocktail (cDC; Il-1β, IL-6, TNFα, PGE_2), or with either prophylactic vaccine cocktail BTI (BCG, Typhim, Influvac) or cocktail BTA (BCG, Typhim, Act-HIB) with PGE_2. (**a**) The expression of maturation markers CD80, CD83, CD86, and CCR7 was measured by flow cytometry. Results are shown as percentage of positive cells. The figures show mean ± SEM of three experiments with different donors. (**b**) IL-12p70 production was measured by ELISA in the supernatant of DC cultures 48h after maturation. Per condition each symbol represents one donor. Means are shown for each maturation cocktail. (**c**) The allostimulatory capacity of the DC was tested in a mixed lymphocyte reaction. Allogeneic peripheral blood leukocytes were co-cultured with differently matured DC and T cell proliferation was measured by incorporation of tritiated thymidine. The graph represents mean ± SEM counts per minute relative to cDC of three experiments with different donors, performed in triplicate. (**d**) DC were electroporated with mRNA encoding gp100. After 3h, gp100 protein expression was determined by FACS analysis. Filled curves show staining with specific antibody; thin-lined curves show the isotype control. Numbers indicate mean fluorescence intensity.

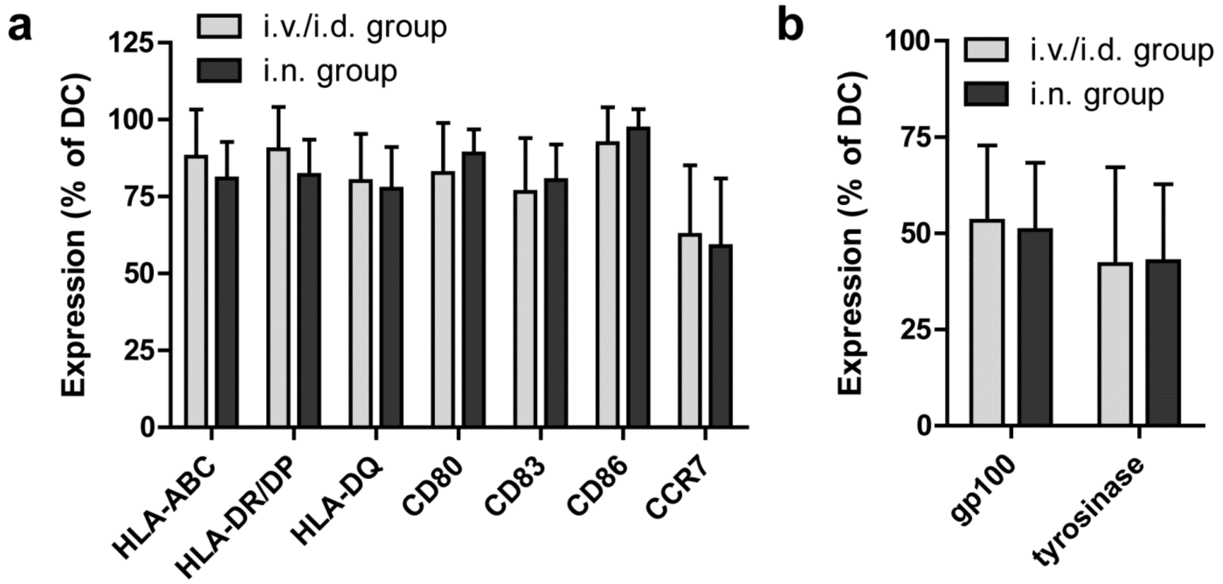

Supplementary Figure 3. Vaccine characteristics of first cycle of all patients

(**a**) Expression of HLA-ABC, HLA-DR/DP, HLA-DQ, CD80, CD83, CD86, and CCR7 was analyzed by flow cytometry. (**b**) Tumor antigen expression by VAC-DC 2-4h after electroporation with mRNA encoding gp100 and tyrosinase. Data are shown as percentage of positive VAC-DC used for the first vaccination. The graphs represent mean ± SD of all patients. Data is shown separately for each administration route; intravenous/intradermal (i.v./i.d.) and intranodal (i.n.) injection of VAC-DC.

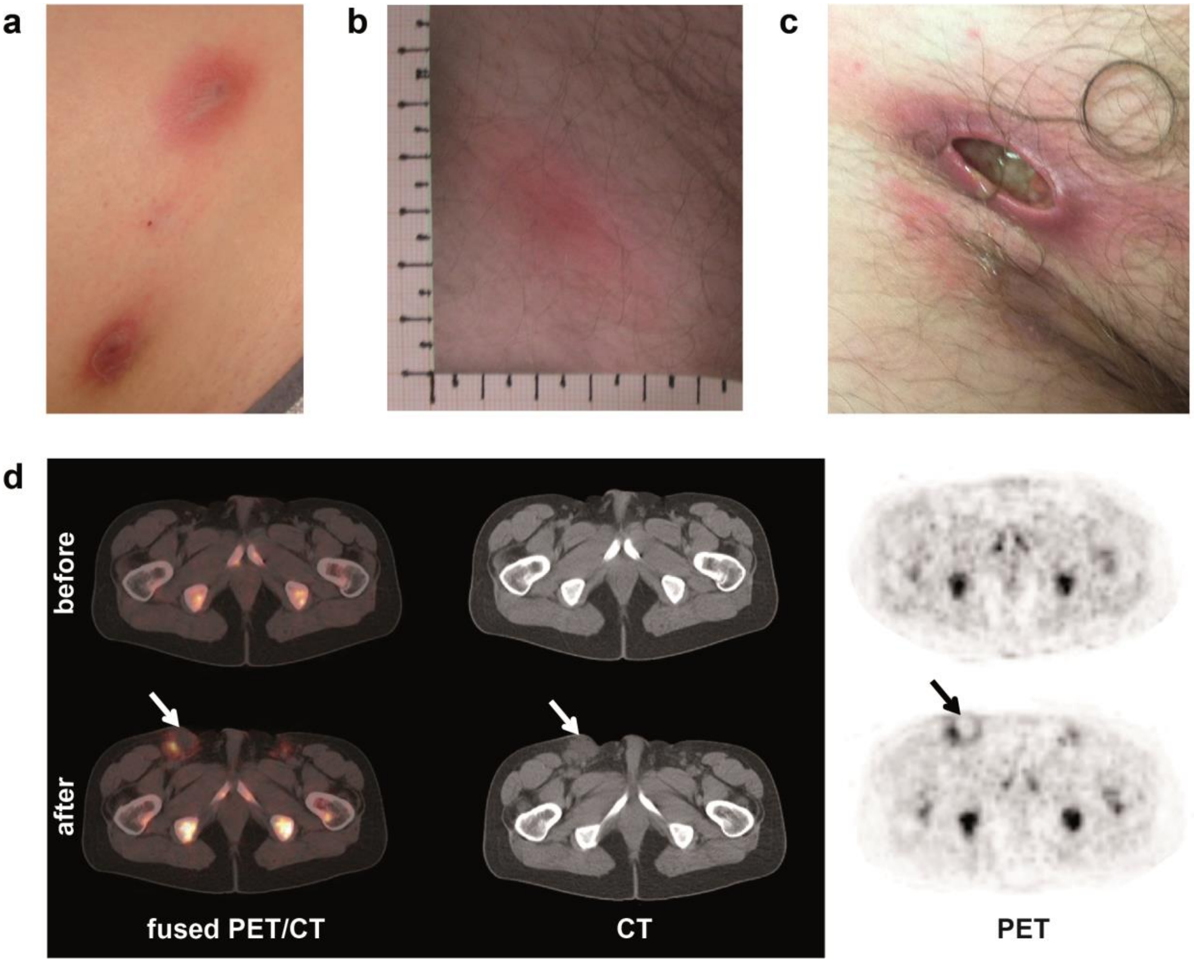

Supplementary Figure 4. Injection site reactions

(**a**) Example of injection site reaction (patient A-11) after intradermal injection with VAC-DC. (**b**) Induration of the injection site after regular flu vaccination (patient A-3). Showing redness over an area with a diameter of 3 to 4 cm. (**c**) Example of injection site reaction (patient B-12) after intranodal injection with VAC-DC, showing substantial swelling of the lymph node (region), redness of the overlying skin and discharge of pus. (**d**) [^{18}F]FLT-PET/CT scan of patient B-12 showing profound lymphadenopathy of the right inguinal lymph nodes after intradermal injection with VAC-DC, with moderately increased [^{18}F]FLT accumulation in the rim and absent tracer accumulation in the centre, suggestive of necrosis (arrow).
